# Cell‐free RNA and fully convolutional dense network‐based early preeclampsia prediction

**DOI:** 10.1002/ctm2.1371

**Published:** 2023-08-15

**Authors:** Zhuo Zhao, Bing Li, Xia Xiao, Jinjun Liu, Wang Zheng

**Affiliations:** ^1^ Key Laboratory of Shaanxi Province for Craniofacial Precision Medicine Research, College of Stomatology Xi'an Jiaotong University Xi'an P. R. China; ^2^ State Key Laboratory for Manufacturing System Engineering Xi'an Jiaotong University Xi'an P. R. China; ^3^ Clinical Research Center of Shaanxi Province for Dental and Maxillofacial Diseases, College of Stomatology Xi'an Jiaotong University Xi'an P. R. China; ^4^ Yanan University Affiliated Hospital Yanan P. R. China; ^5^ Department of Physiology and Pathophysiology, School of Basic Medical Sciences Xi'an Jiaotong University Xi'an P. R. China

Dear Editor,

We propose a fully convolutional dense network (FCDN) model^1^, ^2^ to predict preeclampsia (PE) with circulating cell‐free RNA (cfRNA).^3^, ^4^, ^5^ The Individual Risk Score (IRS) output of the proposed FCDN model contributes to the literature on consistently monitoring the risk of PE, evaluating the effect of prophylactic treatments, and providing accurate as well as rapid screening and diagnosis of PE in populous developing countries with a high incidence of PE, such as China.

PE is a pregnancy‐specific hypertensive disorder and leads to 10.2 deaths per 100 000 pregnancies,^6^, ^7^, ^8^ reaming the second death cause of pregnant women in China. Diagnoses of PE are still regularly missed or delayed and predicting PE in early gestation remains challenging. The trained network is designed to predict PE risk in terms of IRS according to variations in personal cfRNA profiling in early pregnancy (Figure 1A). For the first step, standardized and cleaned cfRNA sequencing data from normal pregnancy (NP) and PE were downloaded from GSE192902^5^ in Gene Expression Omnibus, which were collected ≤12 gestational weeks (gws) or at 13–20 gws. A total of 7160 detected cfRNAs were filtered to select cfRNAs with significant changes that could be used as indicators of PE risk. As illustrated in Figure 1B, we used multiple tests to optimize the parameters of the algorithm, a total of 29 cfRNAs were chosen as PE indicators for samples sequenced at ≤12 gws, and 25 cfRNAs were selected as samples sequenced at 13–20 gws (Table S1).

**FIGURE 1:**
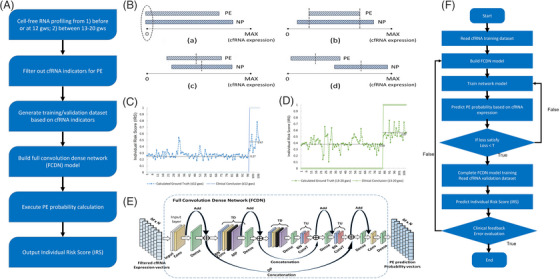
Illustration of study design, data filtration, and model architecture. (A) Study design and prediction mechanism. (B) Principles for data filtration: (a) both PE and control groups contained 0 expression; (b) 100% overlapping domain of cell‐free RNA (cfRNA) expression; (c) larger overlapping domain and smaller mean deviation; (d) significant difference distribution. (C) ground truth Individual Risk Score (IRS) for cfRNA profiling sampled ≤12 gws. (D) ground truth IRS for cfRNA profiling sampled between 13 and 20 gws. (E) Fully convolutional dense network (FCDN) architecture comprised of an input layer, series‐connected residual convolution blocks, and output layers. (F) Procedure diagram of PE prediction based on FCDN. Conv, two‐dimensional convolution; Dense, full connection layer; BN, batch normalization; DP, neuron drop‐out; MP, max‐pooling; ConvT, two‐dimensional deconvolution; TD, transition down module; TU, transition up module.

Given that neural network models require large datasets to perform training, we analyzed the rates of change of real‐world cfRNA profiling data^5^ and generated a synthetic dataset to train the prediction model based on the Gaussian function given below as Equation (1).

(1) $$\begin{array}{c} \left\{\begin{array}{c} x\_train\left[i\right]=rands\left\{Max\left[Max\left(SP[i]\right),Max\left(SN[i]\right)\right],Min\left[Min\left(SP[i]\right),Min\left(SN[i]\right)\right],M\right\}\\ x\_test\left[i\right]=rands\left\{Max\left[Max\left(SP[i]\right),Max\left(SN[i]\right)\right],Min\left[Min\left(SP[i]\right),Min\left(SN[i]\right)\right],Q\right\} \end{array}\right.\\ i\in [1,2,3,\text{…},N] \end{array}$$

where *N* = *s*; rands () is the Gaussian random function, Max () and Min () are the maximum and minimum value functions, respectively, and M and Q are the numbers to be produced.

Therefore, the vector set of cfRNA contributions (y_train and y_test) can be calculated from x_train, x_test, and clinical diagnosis (prior knowledge). This process is performed as given in Equations (2) and (3).

(2) $$\begin{array}{c} \left\{\begin{array}{c} y\_train\left[i\right]=\frac{x\_train[i]-Min(x\_train[i])}{Max(x\_train[i])-Min(x\_train[i])},\;\;if\;\;avg\left(SP\left[i\right]\right)>avg\left(SN\left[i\right]\right)\\ y\_train\left[i\right]=\frac{Max(x\_train[i])-x\_train[i]}{Max(x\_train[i])-Min(x\_train[i])},\;\;if\;\;avg\left(SP\left[i\right]\right)<avg\left(SN\left[i\right]\right) \end{array}\right.\\ i\in [1,2,3,\text{…}N] \end{array},$$

(3) $$\begin{array}{c} \left\{\begin{array}{c} y\_test\left[i\right]=\frac{x\_test[i]-Min(x\_test[i])}{Max(x\_test[i])-Min(x\_test[i])},\;\;if\;\;avg\left(SP\left[i\right]\right)>avg\left(SN\left[i\right]\right)\\ y\_test\left[i\right]=\frac{Max(x\_test[i])-x\_test[i]}{Max(x\_test[i])-Min(x\_test[i])},\;\;if\;\;avg\left(SP\left[i\right]\right)<avg\left(SN\left[i\right]\right) \end{array}\right.\\ i\in [1,2,3,\text{…}N] \end{array},$$

where *N* = *s*, avg () is the average value function. Dataset for FCDN model training and validation is shown in Supplementary Table 2.

In fact, only one clinical diagnosis conclusion was available for any enrolled woman: NP or PE. Therefore, we define that women in the PE group have the maximum IRS = 1 and NP have minimum IRS = 0. Based on the sequencing cfRNA from enrolled women, we can calculate their IRS using Equations (2) and (3). Next, we calculated the IRS at that sampling time. Calculated IRS is regarded as the ground truth for the enrolled women. At a sampling time ≤12 gws, the average of calculated IRS was 0.27 and 0.47 in NP and PE group, respectively (Figure 1C). At a sampling time of 13–20 gws, the average calculated IRS of NP and PE was 0.39 and 0.57, respectively (Figure 1D). The average calculated IRS for the NP group differed notably from that of the PE group (Supplementary Figure 1). The results suggest that the filtered cfRNA indicators work well to distinguish NP from PE and support the application of the proposed model in clinical practice.

Next, we used an FCDN model to perform data regression (Figure 1E). The procedure of FCDN on PE prediction is shown in Figure 1F. In the current study, different datasets were used for model training and validation. The dataset in GSE192902 was divided into Discover Cohort, Validation 1 Cohort and Validation 2 Cohort. For model training, Validation 2 Cohort (87 sets of real‐world cfRNA profiles) and 7913 computer‐generated cfRNA profiles were employed. For model validation, 1000 computer‐generated cfRNA profiles were employed. For the final model validation (application), we used Discover Cohort, and Validation 1 Cohort, which include 215 sets of real‐world cfRNA profiles. A more detailed method for FCND construction, training and validation is shown in the Supporting Information. Through FCDN model training and validation (Figure 2A,B), the loss value (mean absolute error [MAE]) of the probability prediction decreased to 0.027, and an optimized model could then be obtained. To validate the prediction accuracy of the model, we used cfRNA expression from the real world^5^ as the input set x_test for the FCDN model to obtain the FCDN‐based IRS. Furthermore, the FCDN‐based IRS was compared with the ground truth (calculated IRS) calculated from real‐world cfRNA profiling. At a sampling time of ≤12 gws, the tendency and amplitude of the FCDN‐based IRS (prediction results) resembled the ground truth, suggesting the fitting ability of our FCDN model (Figure 2C). The MAE between the prediction result and the ground truth was only 0.032. PE and NP can be separated using averaged FCDN‐based IRS. We also calculated the FCDN‐based IRS for samples enrolled 13–20 gws (Figure 2D). Over the whole scale, the prediction results approximate the ground truth. The MAE between the prediction result and ground truth was only 0.041, indicating that the FCDN model predicted the ground truth well.

**FIGURE 2:**
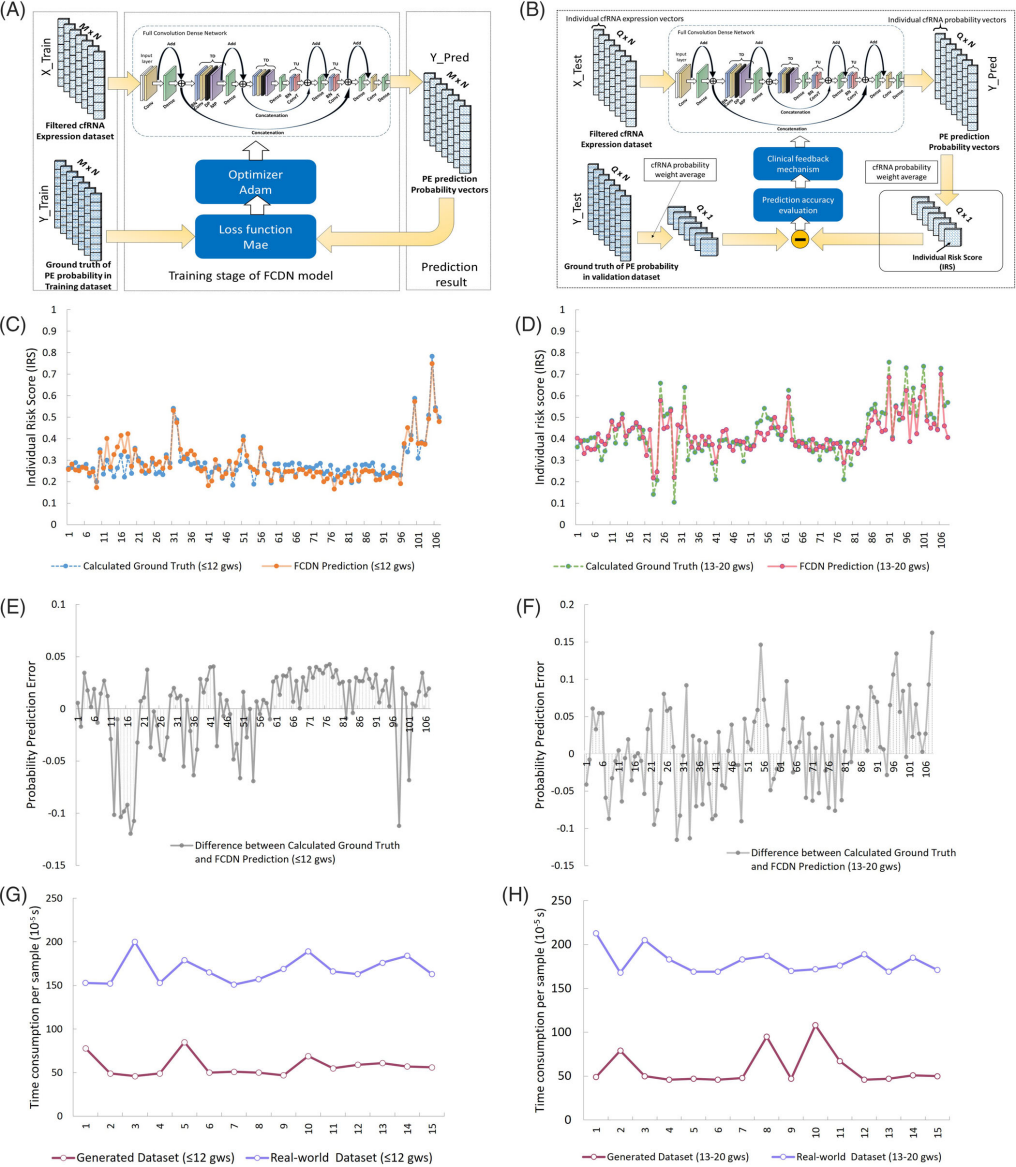
Experimental results. (A) Training strategy; X_Train, cell‐free RNA (cfRNA) expression matrixes in training dataset; Y_Train, corresponding probability vectors that contribute to PE; Y_Pred, The predicted probability vectors that are produced by fully convolutional dense network (FCDN); M × N, there are M profilings in the dataset whose cfRNA indictors (filtered) is 1 × N. (B) Validation strategy; X_Test, cfRNA expression matrixes in validation dataset; Y_Test, corresponding probability vectors that contribute to PE; Y_Pred, the predicted probability vectors that produced by FCDN; Q × N, there are M profilings in the dataset whose cfRNA indictors (filtered) is 1 × N. (C) FCDN‐based Individual Risk Score (IRS) (prediction results) for cfRNA profiling sampled ≤12 gws. (D) FCDN‐based IRS (prediction results) for cfRNA profiling sampled between 13 and 20 gws. (E) Error amplitude of FCDN‐based IRS for cfRNA profiling sampled ≤12 gws. (F) Error amplitude of FCDN‐based IRS for cfRNA profiling sampled between 13 and 20 gws. (G) Processing efficiency for cfRNA profiling sampled≤12 gws. (H) Processing efficiency for cfRNA profiling sampled between 13 and 20 gws. IRS, individual risk score; gws, gestational weeks.

The error amplitude of the FCDN‐based IRS in processing cfRNA samples ≤12 gws is shown in Figure 2E. The maximum value of the absolute error, the peak‐to‐valley (PV) value of the error, and the mean value of the absolute error were 0.12, 0.16 and 0.046, respectively. For samples within 13−20 gws (Figure 2F), the maximum value of the absolute error reached 0.16, and the PV value of the error reached 0.27. The mean absolute error was 0.008. In short, the prediction error for IRS was well‐controlled within a small amplitude, and the FCDN model was able to fit the data well.

We also considered processing efficiency when dealing with numerous datasets collected from population screening. Therefore, the prediction time efficiency was also used as another benchmark to evaluate the method. In this test, the cfRNA profiling samples were fed into the trained FCDN model, and the time required to output an IRS value was recorded. As shown in Figure 2G,H, the results of 15 consecutive experiments showed that the average time required to output an IRS reached 10^−5^ s per sample.

In summary, we employed novel biomarker cfRNAs and an FCDN model to output an IRS to predict PE. The prediction accuracy and computational time of the proposed model reached 0.95 and 10^−5^ s per sample, respectively. The reported method provides a reliable tool for rapid and minimally invasive monitoring of individual PE risk and sheds new light on maternal and neonatal healthcare (Figure 3).

**FIGURE 3:**
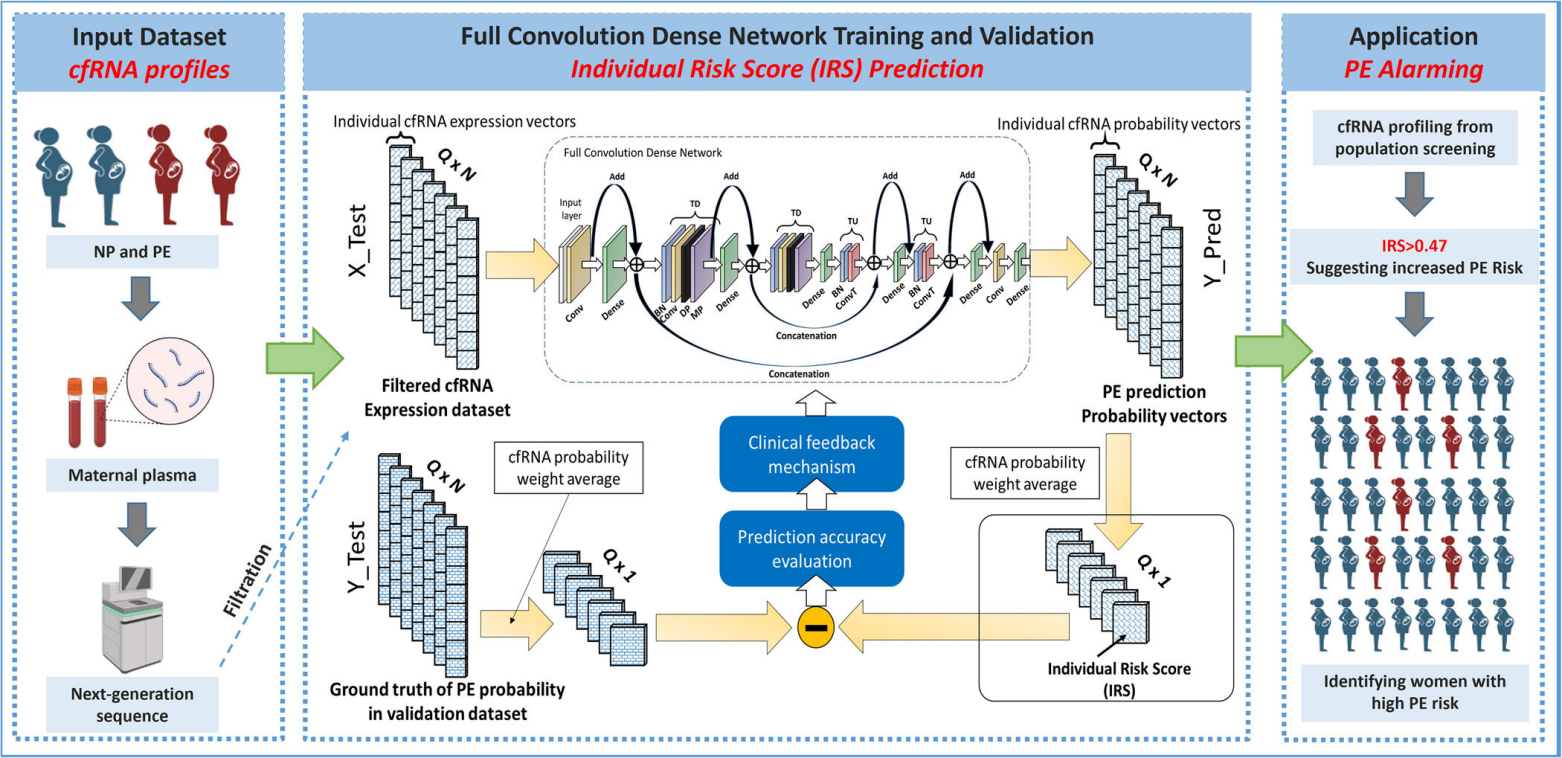
Cell‐free RNA and fully convolutional dense network‐based early preeclampsia prediction. This study developed a deep learning algorithm to evaluate Individual Risk Score (IRS) for pregnant women using cell‐free RNA (cfRNA) profiling; the IRS output of the proposed FCDN model contributes to the literature on consistently monitoring the risk of PE, evaluating the effect of prophylactic treatments, and providing accurate as well as rapid screening and diagnosis of PE in populous developing countries. In current study, different datasets were used for model training and validation. The dataset in GSE192902 were divided into Discover Cohort, Validation 1 Cohort, and Validation 2 Cohort. For model training, Validation 2 Cohort (87 sets of real‐world cfRNA profiles) and 7913 computer‐generated cfRNA profiles were employed. For model validation, 1000 computer‐generated cfRNA profiles were employed. For the final model validation (application), we used Discover Cohort, and Validation 1 Cohort, which include 215 sets of real‐world cfRNA profiles. NP, normal pregnancy; PE, preeclamptic pregnancy; cfRNA, circulating cell‐free RNA. IRS, Individual Risk Score.

## CONFLICT OF INTEREST STATEMENT

The authors declare no conflict of interest.

## FUNDING INFORMATION

This work was supported by the National Natural Science Foundation of China (82071670 and 81771616) to Jinjun Liu, Natural Science Foundation of Shaanxi Province (2023‐JC‐QN‐0954) to Zheng Wang, Funding of Clinical Research Center of Shaanxi Province for Dental and Maxillofacial Diseases (2022YHJB08) to Xia Xiao and Jilin Science and Technology Development Project No. 20210502028ZP.

## Supporting information

Supporting Information

Supporting Information

**Figure S1** This figure shows the calculated IRS of enrolled subjects in NP and in PE at different sampling times. The green line refers to the calculated ground truth (also IRS) at a sampling time of 13–20 gws and the green dot refers to each enrolled subject. The blue line refers to the calculated ground truth (IRS) at a sampling time ≤12 gws and the blue dot refer to each enrolled subject. IRS, Individual Risk Score.

The data and detailed method underlying this article are available in the article and in its online Supplementary Material. The cfRNA employed in the current study was downloaded from the Gene Expression Omnibus (GSE192902).

## Data Availability

Codes and scripts developed for this study are available upon reasonable request.
