# Supplementary material for: Cell‐free RNA and fully convolutional dense network‐based early preeclampsia prediction
Source: Clin Transl Med. 2023 Aug 15;13(8):e1371. doi: 10.1002/ctm2.1371 (PMC10426394; doi:10.1002/ctm2.1371)
Supplement: Supplementary file 2 — Supporting Information [file CTM2-13-e1371-s001.docx]

**Supplementary Table 2. Dataset for FCDN model training and validation**

| Dataset | Dimension | Transformed Dimension | Note |
| --- | --- | --- | --- |
| x_train | [M，N] N=s | [1，N，M] M=8000 | Training dataset (cfRNA expression) |
| y_train | [M，N] N=s | [1，N，M] M=8000 | Training dataset (PE probability) |
| x_test | [Q，N] N=s | [1，N，Q] Q=1000 | Validation dataset (cfRNA expression) |
| y_test | [Q，N] N=s | [1，N，Q] Q=1000 | Validation dataset (PE probability) |
