# Supplementary figures and images for: Cell‐free RNA and fully convolutional dense network‐based early preeclampsia prediction
Source: Clin Transl Med. 2023 Aug 15;13(8):e1371. doi: 10.1002/ctm2.1371 (PMC10426394; doi:10.1002/ctm2.1371)

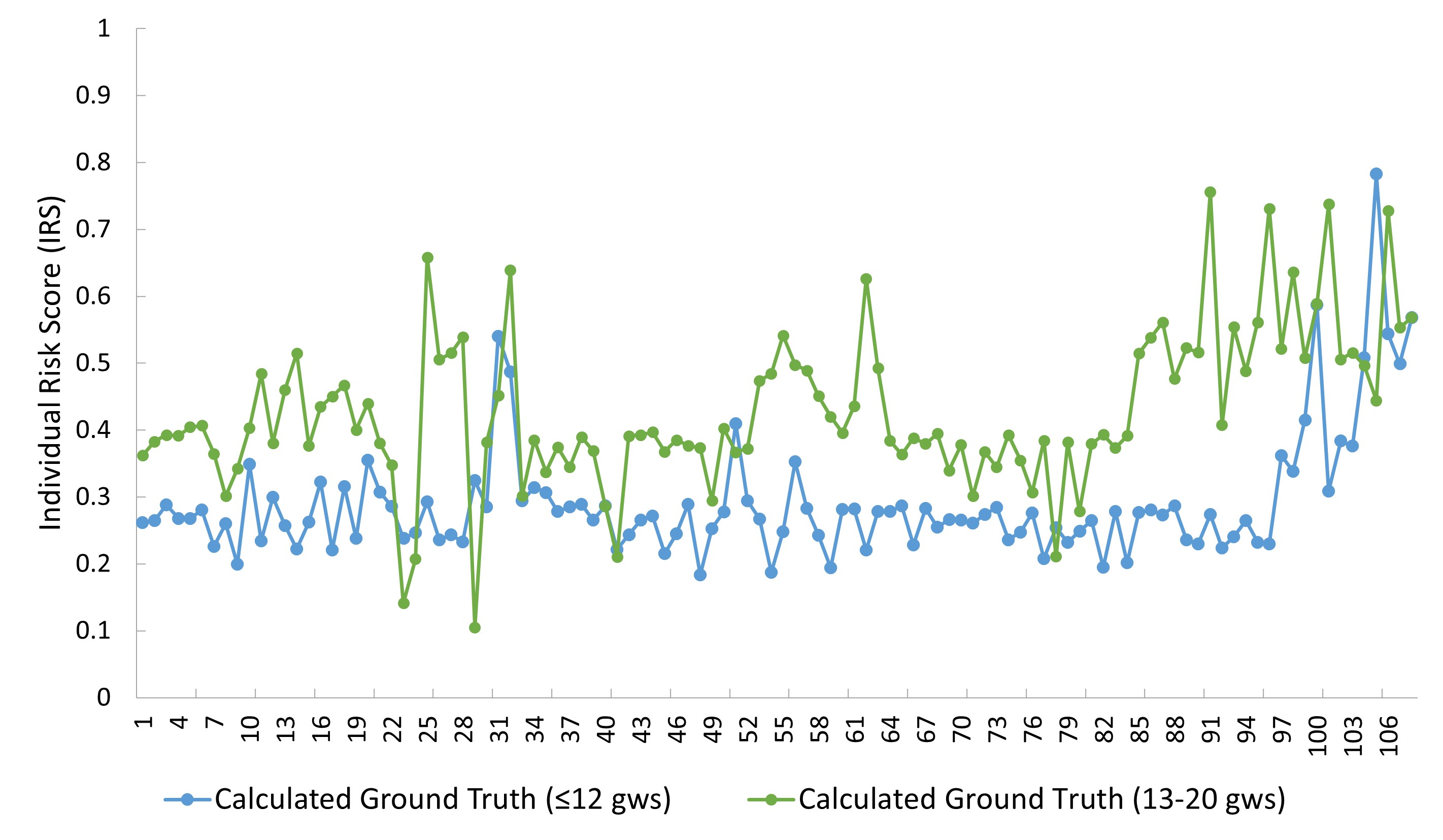

Supplement: Supplementary file 3 — Figure S1 This figure shows the calculated IRS of enrolled subjects in NP and in PE at different sampling times. The green line refers to the calculated ground truth (also IRS) at a sampling time of 13–20 gws and the green dot refers to each enrolled subject. The blue line refers to the calculated ground truth (IRS) at a sampling time ≤12 gws and the blue dot refer to each enrolled subject. IRS, Individual Risk Score. [file CTM2-13-e1371-s004.jpg]
