# Supplementary material for: Cell‐free RNA and fully convolutional dense network‐based early preeclampsia prediction
Source: Clin Transl Med. 2023 Aug 15;13(8):e1371. doi: 10.1002/ctm2.1371 (PMC10426394; doi:10.1002/ctm2.1371)
Supplement: Supplementary file 4 — The data and detailed method underlying this article are available in the article and in its online Supplementary Material. The cfRNA employed in the current study was downloaded from the Gene Expression Omnibus (GSE192902). [file CTM2-13-e1371-s002.docx]

**Materials and Methods**

***Study Design and Prediction Mechanism***

We considered predicting PE risk as a data regression problem, and thus established a mapping relationship between maternal plasma cfRNA profiling and probability vectors. To implement accurate prediction, the initial cfRNA sequencing data must be preprocessed to filter out cfRNA indicators with significant differences between the NP and PE groups. Subsequently, we constructed training and validation datasets for the neural model using the filtered indicators. The trained network is designed to predict PE risk in terms of IRS according to variations in personal cfRNA profiling in early pregnancy (**Figure 1A).**

***Downloading Real-World cfRNA Profiling Data***

Standardized and cleaned cfRNA sequencing data from NP and PE were downloaded from the Gene Expression Omnibus (GSE192902). The diagnosis of PE was defined according to the guidelines provided by the American College of Obstetrics and Gynecology (ACOG), whereas women without complicated pregnancies were defined as a normal control group. None of the included patients had chronic hypertension or gestational diabetes. In addition, we matched the race and ethnicity of the NP and PE groups. Within-cohort (NP versus PE) and across-cohort differences in demographic variables were tested using a two-sided chi-squared test and ANOVA for categorical and continuous variables. Overall, 87 sets of real-world cfRNA profiles were downloaded as training dataset and 215 sets of real-world cfRNA were used as validation dataset.

***Data Filtration***

The data were filtered to select cfRNAs with significant changes that could be used as indicators of PE risk. Only by selecting these indicators successfully can the relationship between multidimensional cfRNA expression profiling and PE risk be established. The preprocessing of cfRNA sequencing data (**Figure 1B**) comprised four key steps, including1) eliminating cfRNAs with zero expression in both NP and PE groups, 2) eliminating cfRNAs with 100% overlapping domains in NP and PE groups, 3) calculating the mean deviation between the distribution domain of cfRNAs in the NP and PE groups, and 4) screening out significant differences in cfRNA according to the overlapping ratio of the distribution domain.

***Dataset Generation and Definition of Individual Risk Score (IRS)***

Given that neural network models require large datasets to perform training, we analyzed the rates of change of real-world cfRNA profiling data and generated a synthetic dataset to training the prediction model. The methods used to construct the dataset are described below.

Before cfRNA filtration, cfRNAs in the NP and PE groups are denoted as CN [cfRNA, normal pregnancy] (**[r, n]**) and CP [cfRNA, pre-eclamptic pregnancy] (**[r, p]**), respectively. A total of 7160 cfRNAs need to be filtered, and “r” denotes the identification number of these cfRNAs, while “n” and “p” represent the identification numbers of participants enrolled in the NP and PE groups.

After filtration and selection, cfRNAs in the NP and PE groups are denoted as SN [selected cfRNA, normal pregnancy] (**[s, n]**) and SP [selected cfRNA, pre-eclamptic pregnancy] (**[s, p]**), respectively. “s” indicates the number of cfRNAs after filtration, the value of which is related to the parameters in preprocessing algorithm.

Based on the filtered cfRNAs, a training dataset is constructed for the proposed FCDN model. The training dataset includes the parameters x_train and y_train, and the validation dataset includes x_test and y_test, where x_train and x_test are cfRNA expression matrices and y_train and y_test are the corresponding probability vectors that contribute to PE. Finally, we denote the mean value of the PE probability vector as IRS. The dataset is composed of two parts, including1) practical cfRNA expression sequencing data from maternal peripheral blood and 2) values randomly generated by a Gaussian function. X_train and x_test are cfRNA expression quantities, the distribution domains of which should follow practical sequencing ranges. The dataset is generated based on the Gaussian function given below as Eq. (1).

$\begin{matrix} \left\{\begin{matrix} x\_train[i]=rands\left\{Max\left[Max\left(SP[i]\right),Max\left(SN[i]\right)\right],Min\left[Min\left(SP[i]\right),Min\left(SN[i]\right)\right],M\right\} \\ x\_test[i]=rands\left\{Max\left[Max\left(SP[i]\right),Max\left(SN[i]\right)\right],Min\left[Min\left(SP[i]\right),Min\left(SN[i]\right)\right],Q\right\} \end{matrix}\right. \\ i\in[1,2,3,\cdots,N] \end{matrix}$ (1)

where *N* = *s*; rands () is the Gaussian random function, Max () and Min () are the maximum and minimum value functions, respectively, and M and Q are the numbers to be produced. Based on the expression and clinical diagnosis (prior knowledge) of SN [s, n] and SP [s, p], we calculate the contribution of each cfRNA to the occurrence of PE or NP. For example, the expression of ENSG00000000460 in the PE group was significantly higher than that in the NP group and reached the maximum expression among all detected cfRNAs. Then, we define that the contribution of ENSG00000000460 to PE is “1”, and vice versa.

Therefore, the vector set of cfRNA contributions (y_train and y_test) can be calculated from x_train, x_test, and clinical diagnosis (prior knowledge). This process is performed as given in Eqs. (2) and (3).

$\begin{matrix} \left\{\begin{matrix} y\_train[i]=\frac{x\_train[i]-Min(x\_train[i])}{Max(x\_train[i])-Min(x\_train[i])}\text{ },\text{ }if\text{ }avg(SP[i])>avg(SN[i]) \\ y\_train[i]=\frac{Max(x\_train[i])-x\_train[i]}{Max(x\_train[i])-Min(x\_train[i])}\text{ },\text{ }if\text{ }avg(SP[i])<avg(SN[i]) \end{matrix}\right. \\ i\in[1,2,3,\cdots N] \end{matrix}$, (2)

$\begin{matrix} \left\{\begin{matrix} y\_test[i]=\frac{x\_test[i]-Min(x\_test[i])}{Max(x\_test[i])-Min(x\_test[i])}\text{ },\text{ }if\text{ }avg(SP[i])>avg(SN[i]) \\ y\_test[i]=\frac{Max(x\_test[i])-x\_test[i]}{Max(x\_test[i])-Min(x\_test[i])}\text{ },\text{ }if\text{ }avg(SP[i])<avg(SN[i]) \end{matrix}\right. \\ i\in[1,2,3,\cdots N] \end{matrix}$ , (3)

where *N* = *s*, avg () is the average value function.

Finally, we obtain x_train, y_train with dimensions [M, N] and x_test, y_test with dimension [Q, N]. Through dimension transformation, these values are reshaped to [1, N, M] and [1, N, Q], respectively. Here, N = s, M=8000, Q=1000, which means that there were 8000 training sample vectors in 1×N and 1000 validation sample vectors in 1×N. IRS = avg (y_test). Dataset for FCDN model training and validation is shown in **Supplementary Table 2.**

***Full Convolution Dense Network (FCDN) Construction***

The FCDN is an optimal structure that has exhibited remarkable performance in multi-object classification and image segmentation as well as predicting and fitting data. We used a FCDN model to perform data regression. Once the relationship between cfRNA expression and PE probability is established, we can determine the PE risk of certain pregnant women based on their cfRNA expression profiles. The structure of the FCDN model comprises an input layer, series-connected residual convolutional blocks, and an output layer (**Figure 1E**). In the FCDN model, BN refers to batch normalization, Conv to two-dimensional convolution, ConvT to two-dimensional deconvolution, ReLu indicates a linear rectification function (activation function) in convolution, MP denotes max-pooling, Dense indicates a fully connection layer, DP indicates a neuron dropout operation, and TU denotes fine feature extraction modules.

The dimension of the model input dataset was (1, N, M), in which there were M samples from pregnant women with 1×N cfRNA expression. The corresponding probability vector was (1, N, M), in which there were M samples with a 1×N probability vector of exhibiting PE.

Principle of incidence prediction based on FCDN.

The cfRNA dataset x_train/x_test was placed into the FCDN model batch by batch from the input layer. The data flow first passes through the convolutional layer for coarse feature extraction at a shallow level.

Then, dense layers and add operations construct a residual network structure, which can recombine multi-dimensional feature maps for fine analysis in latter stages. The FCDN model and fine feature extraction modules (TU) are comprised of four parts, including batch normalization (BN), two-dimensional convolution (Conv) with the ReLU activation function, a neuron dropout operation (DP), and a max-pooling operation (MP). The main function of TU is to accurately capture the variation features of cfRNA in the dataset and perform spatial downsampling processing. Then, the feature maps are decoded after 2-level combination and deconvolution transformations.

Based on the former structure, we also implement a concatenation operation to combine lower- and higher-dimensional features from former and latter layers and improve the fitting capability of the network model. This design can fit the changing relationship between cfRNA expression profiles and IRS of PE and can extract the variation of data in detail.

Finally, using Conv and Dense layers, the feature map tensors from the former level are recombined as the output vector of the model. The output is a (1, N, M)-dimensional probability vector which indicates the IRS of PE, where M is the number of samples and 1×N stands for the PE probability contributions from the N cfRNA index in a single sample.

The procedure of predicting PE based on FCDN (**Figure 1F**) is performed as follows.1) Load the cfRNA training dataset produced by the preprocessing stage with significant difference. 2) Construct FCDN and train the model with the training dataset. 3) After numerous rounds of training, the training stage is complete when loss value between y_pred and y_train satisfies the loss < T (threshold T=0.03). 4) Use the optimal (trained) FCDN model to process validation dataset x_test (with non-overlapping cfRNA data from x_train) and predict IRS. By comparison with the clinical diagnostic conclusion, the prediction results can also be used for accuracy evaluation. If the FCDN-based IRS is similar to the calculated IRS of the clinical diagnostic conclusion, the predicted result can be regarded as true. Otherwise, the training dataset should be corrected based on the clinical diagnostic conclusion and the FCDN model should be retrained to update its parameters.

***Model Training and Validation***

To train our FCDN model, we used 8000 cfRNA profiles, which included 87 sets of real-world cfRNA profiles and 7913 computer-generated cfRNA samples from the validation dataset in a previous study

To validate our FCDN model, 1000 sets of computer-generated cfRNA datasets were used. For the final application, 215 sets of real-world cfRNA profiles from 181 NP and 34 PE from a previous study were used to validate the performance of the model, which can be compared with clinical diagnosis.

To realize PE prediction with higher efficiency and accuracy, the FCDN model should be trained to optimize its parameters after it is constructed. In the training stage, the loss value is denoted as the deviation between the predicted values y_pred and the ground truth y_train. The mean absolute error (MAE) function is adopted as the loss function as defined as Eq. (4).

$MAE=\frac{1}{M}\sum_{i=1}^{M} \left|y[i]-FCDN\left(x[i]\right)\right|$, (4)

where M is the number of samples, y[i] is the incidence probability vector in the dataset, x[i] is the cfRNA expression vector in the dataset, and FCDN ( ) is the proposed prediction method. In the training stage, we adopted the Adam optimizer (**Figure 2E**).

During training, the cfRNA expression dataset x_train is fed into the FCDN model batch-by-batch (batch size = 64). After the prediction, we calculate the MAE loss value between the prediction probability vectors y_pred and the ground truth of the PE probability y_train.

Then, using the Adam optimizer, all the parameters in the FCDN model are updated epoch-by-epoch during the iterative calculation of training. Finally, the training process is terminated under the condition of loss< T (threshold =0.02). In this study, we also design a learning rate decay mechanism to make the learning rate more adaptive to training targets as in Eq. (5).

, (5)

where LR is the model learning rate, loss is the loss value MAE, and attenuation coefficient a=1, b=0, and c=0.5. Obviously, as the loss value decreases, LR is reduced accordingly. The advantages of this strategy are 1) improved stability in the training stage and 2) inhibited oscillation of the loss value in the final training phase.

After training the FCDN model **(Figure 3A)**, we obtain an optimal model that can be used to predict PE based on individual cfRNA profiling. To validate the performance of the trained model, we adopted the validation strategy illustrated in **Figure 3B**.

Validation data (outside the training set) x_test and maternal plasma cfRNA expression profile were fed into the trained FCDN model to produce the predicted PE probability y_pred. In probability vectors, probability values which contributed by each cfRNA expression are taken as the average value and output as the IRS. The prediction error can be evaluated by comparing the predicted probability y_pred with the ground truth. Furthermore, to allow the proposed method to be implemented in clinical application, we corrected the training dataset based on accurate PE diagnoses. Subsequently, we retrained the FCDN model regularly to obtain a more optimal model with higher accuracy.
